# Capturing gene–cell duality in a cat’s cradle

**DOI:** 10.1093/bioinformatics/btaf681

**Published:** 2026-01-11

**Authors:** Anna Laddach, Fränze Progatzky, Vassilis Pachnis, Michael Shapiro

**Affiliations:** Nervous System Development and Homeostasis Laboratory, The Francis Crick Institute, London NW1 1AT, United Kingdom; Nervous System Development and Homeostasis Laboratory, The Francis Crick Institute, London NW1 1AT, United Kingdom; The Kennedy Institute of Rheumatology, University of Oxford, Oxford OX3 7FY, United Kingdom; Nervous System Development and Homeostasis Laboratory, The Francis Crick Institute, London NW1 1AT, United Kingdom; Nervous System Development and Homeostasis Laboratory, The Francis Crick Institute, London NW1 1AT, United Kingdom; Data Science, Brambles, London SW1P 1PL, United Kingdom

## Abstract

**Summary:**

CatsCradle is an R package for single-cell analysis that exploits the duality between cells and the genes they express. Our package provides tools to cluster genes, visualize relationships between them, and to explore relationships between gene clusters (programmes) and cell clusters (cell types).

**Availability and implementation:**

CatsCradle is available freely as an R Bioconductor package (https://bioconductor.org/packages/CatsCradle) and interfaces directly with Seurat (Hao *et al.* 2024) and SingleCellExperiment (Amezquita *et al.* 2020) data structures.

## 1 Introduction

In a scRNAseq dataset duality exists between cells and the genes they express, but this duality is rarely exploited. Cells are typically assigned cell types on the basis of gene expression, and relationships between cell types are visualized using dimensionality reduction techniques, such as UMAP, applied to this gene expression matrix. However, such a cell-centric analysis does not fully take into consideration programmes of gene expression that may cut across cell types.

In our package, CatsCradle (Laddach and Shapiro 2024), we reverse the relationship between genes and cells by transposing the expression matrix. This allows for the elucidation of relationships between genes. Specifically, whereas a standard analysis clusters cells together when they express the same genes, CatsCradle clusters genes together when they are expressed in the same cells. This allows for the identification of genome-wide transcriptional programmes that encompass genes expressed in a coordinate manner. Relationships between genes can be displayed on a gene UMAP (this idea was independently arrived at and exploited in The Human Protein Atlas—proteinatlas.org). In CatsCradle we provide an interactive gene UMAP to facilitate data exploration and interpretation. Although a number of other methods exist for clustering genes, the CatsCradle method, being the direct counterpart of a scRNAseq analysis, is fast and lightweight. Furthermore, CatsCradle functionality can be used to visualize gene modules produced by other software.

Here, we first present methods for clustering and displaying genes in two dimensions and subsequently explore the relationship between cell types and gene expression programmes. For this, we apply CatsCradle, alongside other software for identifying gene modules, to a single cell RNA-seq dataset we reported recently which describes gene expression changes in the muscularis externa of mouse intestine resulting from *Heligmosomoides polygyrus* (*H. polygyrus*) infection. Our analysis reveals the statistically significant co-localisation of genes that belong to the same functional gene sets on this UMAP, including those found to play a key role in the cellular responses to *H. polygyrus*, and for the up-regulation of genes on a per-cell type basis. Further, we show that the function of a gene strongly correlates with those of its neighbouring genes.

Please note that CatsCradle contains methods for studying the relationships between genes and for studying spatial transcriptomics data. We only exposit the former here.

## 2 Results

**Figure 1.**
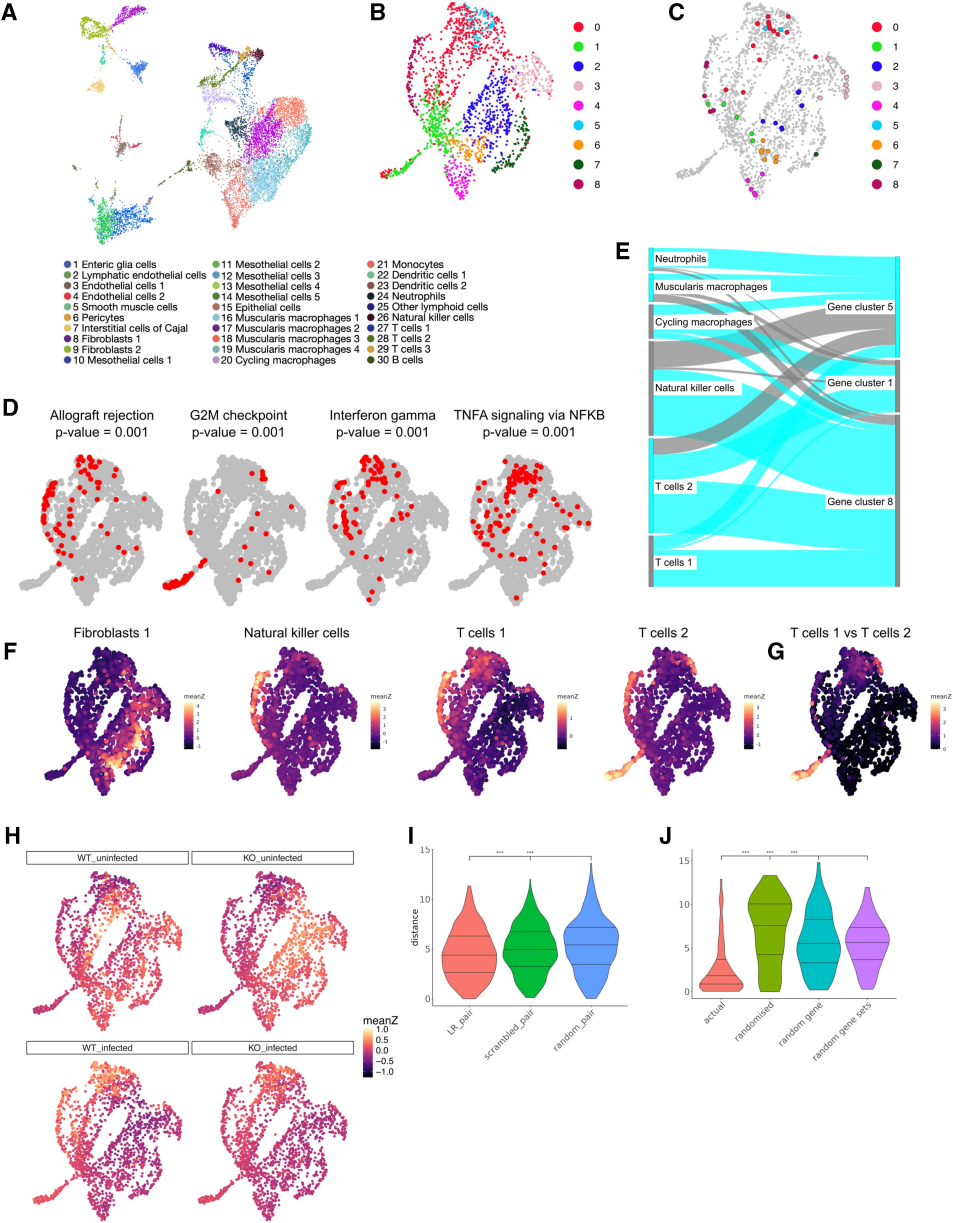
(A) A UMAP of cells. Cells are colored according to cell type. Reproduced from Progatzky *et al.* (2021). (B) A UMAP of genes. Here we use the 2000 most variable genes and cluster them using the Louvain algorithm. (C) As in B with genes encoding transcription factors highlighted. (D) Gene UMAPs with genes from selected Hallmark gene sets highlighted. (E) Sankey diagram showing relationships between selected gene clusters and cell clusters. (F) Gene UMAP coloured by mean *z*-score for expression of each of the 2000 most variable genes in selected cell types. (G) Gene UMAP coloured by absolute value of difference of mean *z*-score for TCells1 and TCells2. (H) Gene UMAP coloured by mean z-score for expression of each of the 2000 most variable genes in different conditions. Here WT and KO refer to control mice Sox10CreERT2; Ifngr2fl/+ (Ifngr2CTRL) and Sox10CreERT 2; Ifngr2fl/fl (Ifngr2ΔEGC) mice respectively. (I) Violin plot showing observed UMAP distances between ligand-receptor pairs (red), randomized ligand-receptor pairs (green), and random gene pairs (blue). (J) Violin plot showing observed UMAP distances between pairs of genes encoding members of actual IntAct complexes, in comparison to randomized distances: the UMAP distance of permuted pairs, the UMAP distance for the first gene of each pair to a randomly chosen gene, and the UMAP distance between pairs derived from randomly chosen gene sets of the same size as our subset complexes.

Data from Progatzky *et al.* (2021) was analysed using CatsCradle. Of note, the dataset was designed to investigate the role of interferon-gamma receptor (Ifngr) signalling within enteric glial cells of the intestinal tunica muscularis during *H. polygyrus* infection. Therefore it contains data both for glial specific Ifngr2 knockout mice $\mathrm{Sox}10Cre^{\mathrm{ERT}2};\mathrm{Ifngr}2^{fl/fl}$ ($\mathrm{Ifngr}2^{\Delta \mathrm{EGC}}$) and control mice $\mathrm{Sox}10Cre^{\mathrm{ERT}2};\mathrm{Ifngr}2^{fl/+}$ ($\mathrm{Ifngr}2^{\mathrm{CTRL}}$). Clusters from Progatzky *et al.* (2021) are depicted on the cell UMAP in Fig. 1A. Analysis, using CatsCradle, gives rise to the gene UMAP in Fig. 1B. Here the top 2000 most highly variable genes across the dataset have been analysed. This is based on the assumption that such genes contain useful biological information, whereas genes with low levels of variability may represent random noise (Luecken and Theis 2019). Genes are clustered into programmes using the Louvain algorithm (UMAP colours, Fig. 1B). Interestingly, most clusters contain several transcription factors (Shannon and Richards 2024), suggesting that these might play a role in the coordinate regulation of gene expression within a cluster (Fig. 1C).

### 2.1 Functional gene sets colocalise on gene UMAP

The question arises as to whether genes involved in the same biological processes/pathways colocalise in gene UMAPs. To investigate this, we first turned to the Hallmark gene sets (Liberzon *et al.* 2015) and intersected each of these sets with the 2000 genes shown. We then plotted these on the gene UMAP and in many cases observed clear co-localisation (see Fig. 1D, Fig. 1, available as supplementary data at *Bioinformatics* online). In order to determine a *P*-value for the geometric clustering of these points we used median complement distance (mcd, see Methods section, Supplementary information, available as supplementary data at *Bioinformatics* online). Across the 50 Hallmark gene sets, 32 show *P*-values ≤ 0.05 (Fig. 2A, available as supplementary data at *Bioinformatics* online); more than expected by chance (*P* ≤ 2.2e-16; binomial test). Amongst gene sets with the lowest *P*-values are *G2M checkpoint*, *interferon gamma response* and *allograft rejection*. Terms that do not show significant clustering include those that are unlikely to be relevant to this dataset, e.g. *pancreas beta cells*, and those which may operate in many cell types, e.g. *fatty acid metabolism*. Put differently, it can be inferred that pathways with significant *P*-values may be of high biological relevance to the tissue or condition being studied.

### 2.2 Functional gene sets give insight into gene clusters

We then studied the relationship between gene clusters and the Hallmark gene sets. Gene set overrepresentation shows strong overlap between certain gene clusters and Hallmark gene sets (Liberzon *et al.* 2015) suggesting that gene clusters reflect gene function (Fig. 2B, available as supplementary data at *Bioinformatics* online). Of note, cluster 1 is highly enriched for genes associated with cell cycle terms, such as *G2M checkpoint* and *mitotic spindle*, whereas cluster 8 is enriched for immune terms such as *allograft rejection* and *Il2 Stat5 signalling*. Cluster 5 also has an immune character, which is however distinct to cluster 8, with enriched terms including *interferon-gamma response* and *TNFA signaling via NFKB*. Relating gene clusters to cell types shows that whilst some programmes are cell type-specific others are active in multiple cell types. For example the cell cycle-associated cluster 1 is expressed by several cell types (such as CyclingMacrophages and the TCells2), whereas cluster 8 is specific to NK and T cells (Fig. 1E, Fig. 3, available as supplementary data at *Bioinformatics* online). Interestingly, the Il2 Stat5 signalling pathway associated with this cluster is known to play key roles in T cell biology (Jones *et al.* 2020). Although allograft rejection superficially appears distinct to *H. polygyrus* infection, both involve antigen presentation and T cell responses. Taken together this suggests that helminth infection induces gene expression changes involved in T cell mediated immunity.

### 2.3 CatsCradle can be used to explore gene modules and regulons derived from other software

We ran several algorithms for detecting gene modules (Delile *et al.* 2019, Feregrino and Tschopp 2022, Yerly *et al.* 2025) on our dataset and visualized the results using the CatsCradle gene UMAP (Fig. 3, available as supplementary data at *Bioinformatics* online). It can clearly be seen that there is a lack of consensus between these methods, both in cluster assignment and the number of total genes assigned to a cluster. In spite of this, all show significant clustering on Gene UMAP. Given that the Hallmark terms *G2M checkpoint*, *allograft rejection*, *interferon-gamma response* and *TNFA signaling via NFKB* show some of the lowest (i.e. strongest) *P*-values for clustering in gene UMAP space, we reasoned that enrichment of these terms in gene modules reflects a biologically informative clustering of genes. Here, we found that CatsCradle modules gave the smallest *P*-values for *allograft rejection* (2.26E-13) and *TNFA signaling via NFKB* (3.75E-14), whereas scWCGNA gave the smallest *P*-value for *interferon-gamma response* (3.05E-5) [closely followed by Catscradle (4.20E-5)] and geneNMF gave the smallest *P*-value for *G2M checkpoint* (9.63E-29) (Fig. 4, available as supplementary data at *Bioinformatics* online, Tables 1–4, available as supplementary data at *Bioinformatics* online). More broadly, all methods gave clusters that showed enrichment for immune terms, however scWCGNA failed to produce a cluster enriched in cell-cycle associated processes (Fig. 4, available as supplementary data at *Bioinformatics* online, Tables 1–4, available as supplementary data at *Bioinformatics* online). Moreover, CatsCradle showed the fastest run time (Table 5, available as supplementary data at *Bioinformatics* online). Taken together, CatsCradle is both fast and reliably detects biologically informative gene modules, but can also be integrated with other methods to enhance analysis.

A similar, but distinct task, is the inference of regulons, that is linking transcription factors to their inferred target genes. While CatsCradle detects co-expression and not mechanism, it can be used to visualize such regulons, inferred using pyScenic (Aibar *et al.* 2017, Van de Sande *et al.* 2020), and to show the relationships between them (Fig. 5, available as supplementary data at *Bioinformatics* online). The overwhelming majority of inferred regulons show highly significant clustering in gene UMAP space. These mappings give a quick way of comparing these regulons to other information, e.g. experimental condition (Fig. 1H), Hallmark gene sets or the up-regulation of genes on a cell-type basis (Figs 1 and 5, available as supplementary data at *Bioinformatics* online).

### 2.4 Cell types have distinct gene expression landscapes

Next, we considered how the 2000 most variable genes are expressed in each of the cell clusters. We started by computing the *z*-score for the expression of each gene across all cells. We then found the average *z*-score for each gene within each cell cluster. Plotting the average *z*-scored expression level on gene UMAP Fig. 1F shows that genes with similar expression levels within a cell type tend to group together spatially on the gene UMAP. Of note, immune cell types tend to show highest mean z-scores in the top left half of the UMAP (corresponding to gene clusters 0, 5, 8), with T cells and NK cells highlighting the region corresponding to cluster 8, whereas non-immune cell types tend to show highest expression levels in the bottom right part of the UMAP (gene clusters 2–4,6,8). Additionally, cycling populations (e.g. cycling macrophages) show high z-scores in the “tail” of the UMAP, corresponding to gene cluster 1 (Fig. 1F). Similar plots are given for all cell clusters in Fig. 6, available as supplementary data at *Bioinformatics* online. We further used Moran’s I (Moran 1950) to measure the spatial autocorrelation of the mean gene *z*-score data for each cell cluster. For each cell cluster, values of Moran’s I between 0.129 and 0.823 were obtained (Fig. 7A, available as supplementary data at *Bioinformatics* online), with *P*-values ≤0.001 as found by permutation testing, further demonstrating that cell type-specific genes tend to colocalise in UMAP space. One can also interrogate differences in gene expression landscapes between cell types of interest. Figure 1G depicts the absolute value for the difference in mean z-score between TCells1 and TCells2; highlighting that the key difference is in the cell cycle associated “tail” of the UMAP.

### 2.5 Gene expression landscapes reflect experimental conditions

We went on to perform a similar analysis where we computed the mean gene *z*-score per condition (see Fig. 1H). A shift in the transcriptomic landscape can be seen 7 days post *H. polygyrus* infection, with the highest mean z scores towards the upper left region of the plot (gene clusters 0, 5, 8), which corresponds to a region rich in genes expressed by immune populations. Interestingly, even at homeostasis the $\mathrm{Ifngr}2^{\Delta \mathrm{EGC}}$ mice have a transcriptional landscape distinct from $\mathrm{Ifngr}2^{\mathrm{CTRL}}$ mice, with a subset of genes from the immune-related cluster 0 demonstrating lower mean z-scores. This is in line with the observation that abrogation of IFNγ signalling in enteric glia in naïve $\mathrm{Ifngr}2^{\Delta \mathrm{EGC}}$ mice is associated with signs of inflammation in the tunica muscularis. Finally, although genes in clusters 0 and 5 are also “activated” upon *H. polygyrus* infection in the $\mathrm{Ifngr}2^{\Delta \mathrm{EGC}}$ mice, it appears that gene cluster 8, characteristic of T cells/NK cells, fails to be activated. This is consistent with the fact that infected $\mathrm{Ifngr}2^{\Delta \mathrm{EGC}}$ mice show a diminished IFNγ response and less CD8+ T cells. Taken together, these results agree with findings from Progatzky *et al.* (2021), which show that glial specific IFN-γ signalling is central to both intestinal homeostasis and response following infectious challenge.

### 2.6 CatsCradle infers gene function

We next set out to investigate whether the genes in our UMAP tend to share functional annotations with their immediate neighbours. To this end, we computed a nearest neighbour graph using the 20 nearest neighbours of each gene in UMAP coordinates. For each annotated gene, we computed the total number of times it shares an annotation with a neighbouring gene. This gave 3682 total agreements, shown by the red line in Fig. 7B, available as supplementary data at *Bioinformatics* online. Note that a gene may have multiple annotations. For example, Abca1 appears in 5 Hallmark gene sets. We then computed similar results based on 1000 randomizations of the nearest neighbour graph using the CatsCradle function randomiseGraph(). This produced the histogram shown in Fig. 7B, available as supplementary data at *Bioinformatics* online, which has a mean of 3023.27 and standard deviation of 81.01. In particular, the actual number of agreements is 8.13 standard deviations above the mean. This suggests that CatsCradle can be used to predict the functions of genes with unknown function.

### 2.7 Ligands and their receptors are close in gene UMAP space

Next, we investigated the relationship between ligands and their receptors on gene UMAP. We used the Nichenetr (Browaeys *et al.* 2020) ligand receptor networks as a listing of ligand-receptor pairs and restricted these to pairs in our dataset, as shown in Fig. 7C, available as supplementary data at *Bioinformatics* online.

We then compared three sets of UMAP distances. The first consisted of the UMAP distances between these ligand-receptor pairs. The second consisted of distances between the ligands and randomized receptors and the third consisted of distances between random gene pairs. The distances between ligands and their receptors were significantly smaller than either of the other two sets with a *P*-value ≤ 0.001 in both cases (Fig. 1I). This suggests that autocrine signalling and intra-cell population paracrine signalling are highly active within this dataset. It could also be taken to suggest that communication might preferentially occur between cell types with more similar transcriptomes. Perhaps surprisingly, distances between ligands and random receptors appear to be larger than distances between random genes.

### 2.8 Subunits of complexes are close in gene UMAP space

Finally, we tested the hypothesis that genes encoding subunits of protein complexes co-localise in the gene UMAP. We used the IntAct resource (Del Toro *et al.* 2022) to identify proteins that form complexes, and converted them to their encoding genes using BioMart (Durinck *et al.* 2005). We then compared four sets of UMAP distances: the UMAP distance for each of these actual pairs, the UMAP distance these pairs after randomizing the second partners in these pairs, the UMAP distance for the first gene of each pair to a randomly chosen gene, and the UMAP distance between pairs derived from randomly chosen gene sets of the same size as our subset complexes. Results show that genes whose protein products are known to form complexes are significantly closer on the UMAP than expected by chance (Fig. 1J), further suggesting that functional relationships are encoded in the gene UMAP.

## 3 Discussion

Our R package CatsCradle exploits the duality between cells and genes. It does this by transposing the gene expression matrix thus swapping the roles of cells and genes. Consequently, the techniques usually used to study cells (e.g. Louvain clustering, UMAP) now apply to genes. Furthermore, we have shown that the resulting analyses encode meaningful biological information. This is revealed in data from (Progatzky *et al.* 2021) in the UMAP locations of Hallmark functional gene sets, gene expression patterns on a per cell type and per condition basis, the relations between ligand-receptor pairs and in the co-location of gene annotations. We have also shown that gene clusters calculated using CatsCradle compare favourably to gene clusters calculated using several established methods. Moreover, the lightweight, fast nature of CatsCradle allows for the quick exploration of data, and for the user to interrogate different clustering resolutions. CatsCradle also provides useful functionality to visualize and explore modules produced using different software.

Of note, for any given application of CatsCradle a specific dataset is utilized. However, while individual cells do not persist across different datasets, genes do. For example, two genes that are coexpressed and cluster together in one dataset might not be coexpressed in another—perhaps because these datasets are derived from different tissues or reflect different treatments. This suggests that the application of CatsCradle to predict gene function might give tissue/condition-specific results. However, the high degree of clustering of the Hallmark gene sets in our current dataset implies that some of the spatial relationships between genes observed might be consistent and widespread across datasets. We believe further investigation of the gene relationships across tissues, conditions and even species presents a promising and important avenue for future research that will enable the prediction of gene function for unknown genes and uncover how gene programmes orchestrate cell functions and mediate interactions within tissue niches across species.

## Supplementary Material

btaf681_Supplementary_Data

## Data Availability

Previously generated data used in this manuscript is available on GEO under the accession GSE182506.
