# Supplementary material for: Capturing gene–cell duality in a cat’s cradle
Source: Bioinformatics. 2026 Jan 11;42(1):btaf681. doi: 10.1093/bioinformatics/btaf681 (PMC12831930; doi:10.1093/bioinformatics/btaf681)
Supplement: btaf681_Supplementary_Data [file btaf681_supplementary_data.zip › 09-Jan-2026_072017_CatsCradleSupplementary.pdf]

Supplementary information

Median complement distance

We would like a method for measuring the geometric clustering of a subset within a given set. The measure we propose here detects the tendency of points to "stick together" rather than their tendence to be confined to a particular region. It might therefore be better thought of as curdling rather than clustering.

Suppose we have a finite set $S \subset \mathbb{R}^N$ and a set $X \subset S$. We would like a measure of the degree to which the points of X are spatially clustered within S. Put differently, we would like to know whether the points of X are evenly spread throughout S or if they have a tendency to "stick together". If they are spread out randomly in S, then we would expect most points of $S \setminus X$, the complement of X in S to be relatively close to some element of X. If, on the other hand, the points of X are clustered together, then we would expect a fair portion of the points of $S \setminus X$ to be relatively far from X. This suggests the following measure. Take $\widehat{S} = S \setminus X$ to be the complement of X in S. For each $s_i \in \widehat{S}$ we take $d_i = d(s_i, X)$ to be the distance from s_i to the nearest element of X. We take the *median complement distance* $\mathrm{mcd}(S, X)$ to be the median of these d_i. A small value for $\mathrm{mcd}(S, X)$ means that X is well spread out among the points of S. Conversely, a large value means that the points of X "stick together" thereby decreasing their overall coverage of the set S.

In order to compute a p-value for the geometric clustering of the points of X in S, we compute $m = \mathrm{mcd}(S, X)$ and then randomly choose subsets $X_i \subset S$ where each of these has the same number of points as X. Computing $m_i = \mathrm{mcd}(S, X_i)$ for each of these random sets X_i allows us to give a p-value for the geometric clustering of X in S. If m is greater than almost all of the m_i, we have detected significance for the clustering of X.

Note that a significant p-value for geometric clustering does not necessarily imply that all the points of X are near each other. Rather, it measures the "stickiness" of the subset, i.e., the tendency of points in the subset to stick together. We can imagine a set S of 2000 points with a subset X of 100 points. These latter points might be located in 10 widely spread locations in S but with the points in each of these 10 locations tightly clustered. This will still result in significant geometric clustering.

Overlap of gene clusters and Hallmark gene sets

To compute p-values for the overlap of gene clusters and Hallmark gene sets, we used a one-sided Fisher's exact test. Taken together, the Hallmark gene sets contain 4394 distinct genes. Together with the 2000 genes of the gene Seurat object, this gives 5420 distinct genes and we took these as our background.

Calculation of gene modules using other software

Gene modules were calculated using scWCGNA 1.0.0 (Feregrino and Tschopp, 2022), GeneNMF 0.8.0 (Yerly et al., 2025) and Antler (Delile et al., 2019) packages in R, using the top 2000 most highly variable genes as input. The first step in the calculation of scWCGNA modules is the calculation of pseudocells (this decreases the size of the data allowing for a reasonable run time); here, the command calculate.pseudocells(s.cells = seurat.obj, seeds=0.2, nn = 10, reduction = "pca", dims = 1:10) was used. scWCGNA modules were then calculated using the run.scWGCNA function with default parameters. GeneNMF was run using the *multiNMF* function to perform non-negative matrix factorization on each sample individually, over a range of target NMF components (k), with assay set to "integrated". The *getMetaPrograms* function was then used to calculate gene programmes with nMP (the number of meta programmes) set to 10, and weight.explained set to 0.05. For the calculation of Antler modules, raw counts were normalised using the command antler$normalize(method = "MR"). Gene modules were then caculated using the command antler$gene_modules$identify(name = "unbiasedGMs", corr_t = 0.5, corr_min = 5, mod_min_cell = 10, mod_consistency_thres = 0.5, process_plots = TRUE), where corr_t is the Spearman correlation threshold, corr_min is the minimum number of genes a gene must correlate with, mod_min_cell is the minimum number of cells that express a module and mod_consistency_thres is the ratio of expressed genes among "positive" cells.

Supplementary Table 5 shows the runtimes for gene module calculation for these algorithms and CatsCradle. Of note, CatsCradle, which utilises Seurat functionality to detect gene modules, has the fastest run time. Although all three algorithms have a reasonable run time on this relatively small dataset, we believe the faster run time of CatsCradle offers greater advantages when applied to larger datasets.

Calculation of regulons

Regulons were calculated using the pySCENIC software (Aibar et al., 2017; Van de Sande et al., 2020), using the pyscenic-0.12.1 singularity image, and counts for the top 2000 most highly variable genes as input. The command *pyscenic grn* was used to build a gene regulatory network, followed by *pyscenic ctx* to infer regulons, with the --mask_dropouts option, and *pyscenic aucell* to quantify regulon activity with cells.

Moran's I

We used the implementation of Moran's I (Moran, 1950) in the CatsCradle (Laddach and Shapiro, 2024) function computeMoransI(). Moran's I depends on a matrix W of weights giving the proximity of distinct points in the data set. For genes i and j, $i \neq j$, we take $W_{ij} = 1$ if i and j are neighbours in the nearest neighbour graph and $W_{ij} = 0$ otherwise. We performed this computation using the nearest neighbour graph computed using the 20 nearest neighbours in the gene UMAP coordinates. In order to compute p-values, we performed permutation testing where we held the z-score values per gene constant and randomised the nearest neighbour graph using the CatsCradle function randomiseGraph(). For each cell type, this produced a p-value for Moran's I of less than 0.001.

Colocalization of complexes

We used the listing of complexes at

https://ftp.ebi.ac.uk/pub/databases/intact/complex/current/complextab/

specifically, the M. musculus file 10090.tsv. This gives a listing of 747 complexes consisting of 1557 distinct species. We used biomaRt (Durinck et al., 2005) to convert UniProtKB to gene names. This produced 1310 distinct species. Of these 130 are found in our gene Seurat object. This produced a list of 56 complexes which contain at least two distinct genes from our gene Seurat object. This, in turn, produced a list of 65 distinct pairs of genes where each pair of genes is found in a common complex. We then computed a) the UMAP distance for each

of these actual pairs, b) the UMAP distance these pairs after randomising the second partners in these pairs, c) the UMAP distance for the first gene of each pair to a randomly chosen gene, and d) the UMAP distance between pairs derived from randomly chosen gene sets of the same size as our subset complexes.

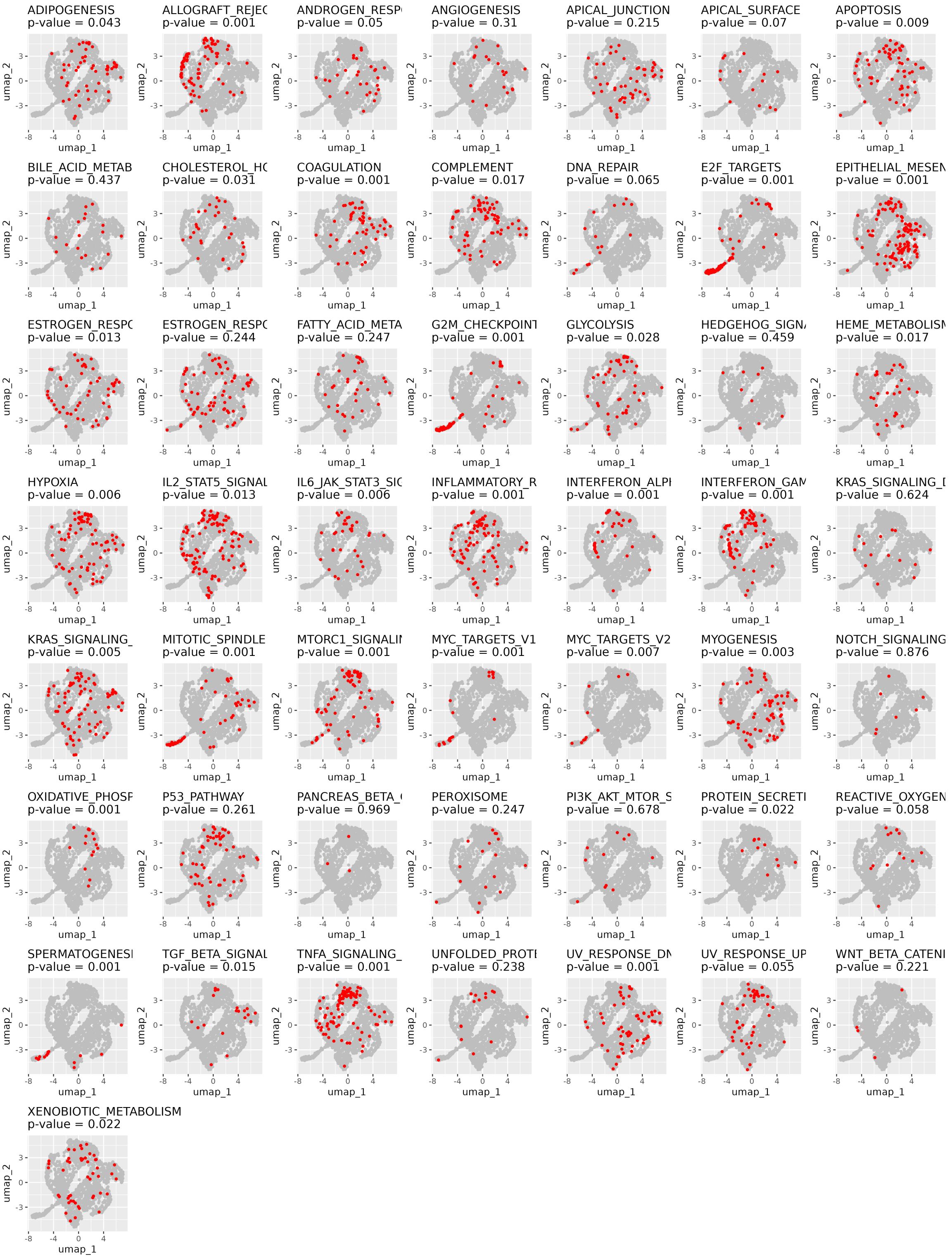

Supplementary Figure 1. For each hallmark gene set we display the overlap of that set with the genes of our gene UMAP along with their mcd p-value. Among the most significant gene sets are those associated with immune response and with proliferation.

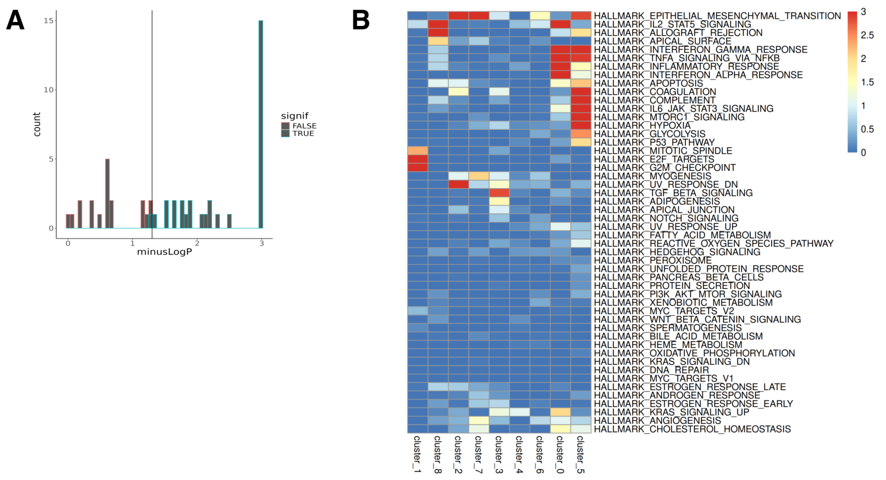

Supplementary Figure 2. **A)** Histogram of mcd p-values for Hallmark gene sets, subset to the genes of our gene UMAP. **B)** Heatmap showing minus log10 of p-values for overlap between gene clusters and Hallmark gene sets. We have imposed a minimum p-value of 0.001 for visual clarity. **C)** Histogram showing Moran's I for co-localization of z-scores per gene per cell cluster.

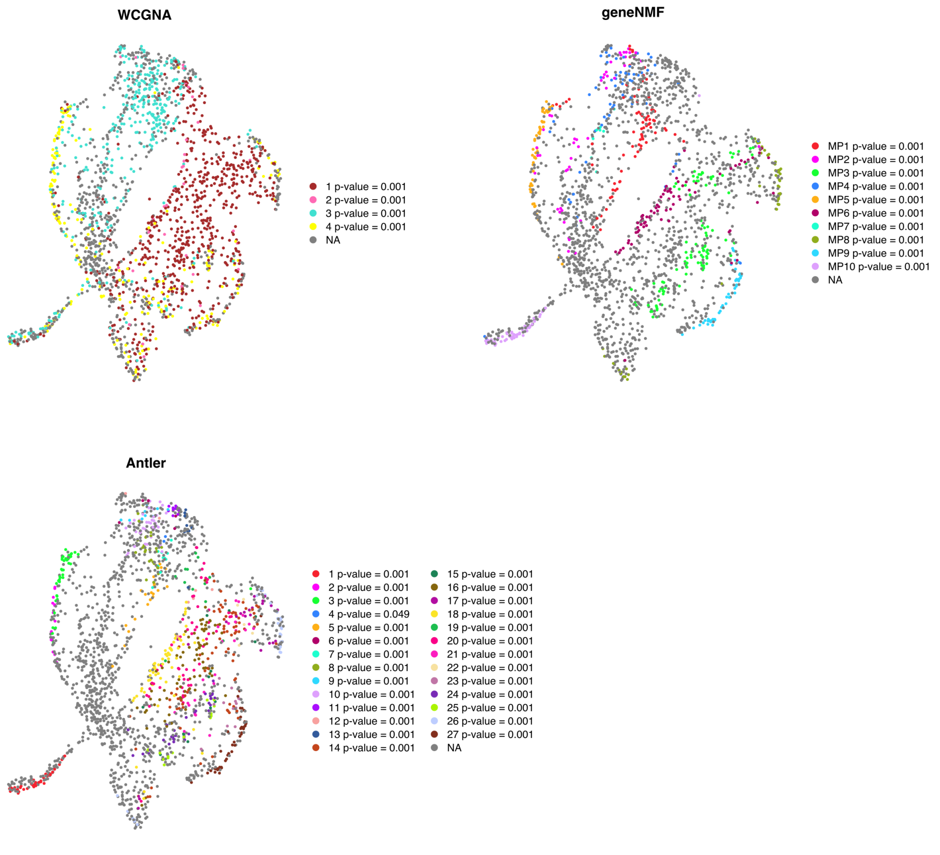

Supplementary Figure 3. Gene modules calculated using the scWCGNA, GeneNMF and Antler software displayed on the CatsCradle gene UMAP. The mcd p-value for each module is shown in the legend.

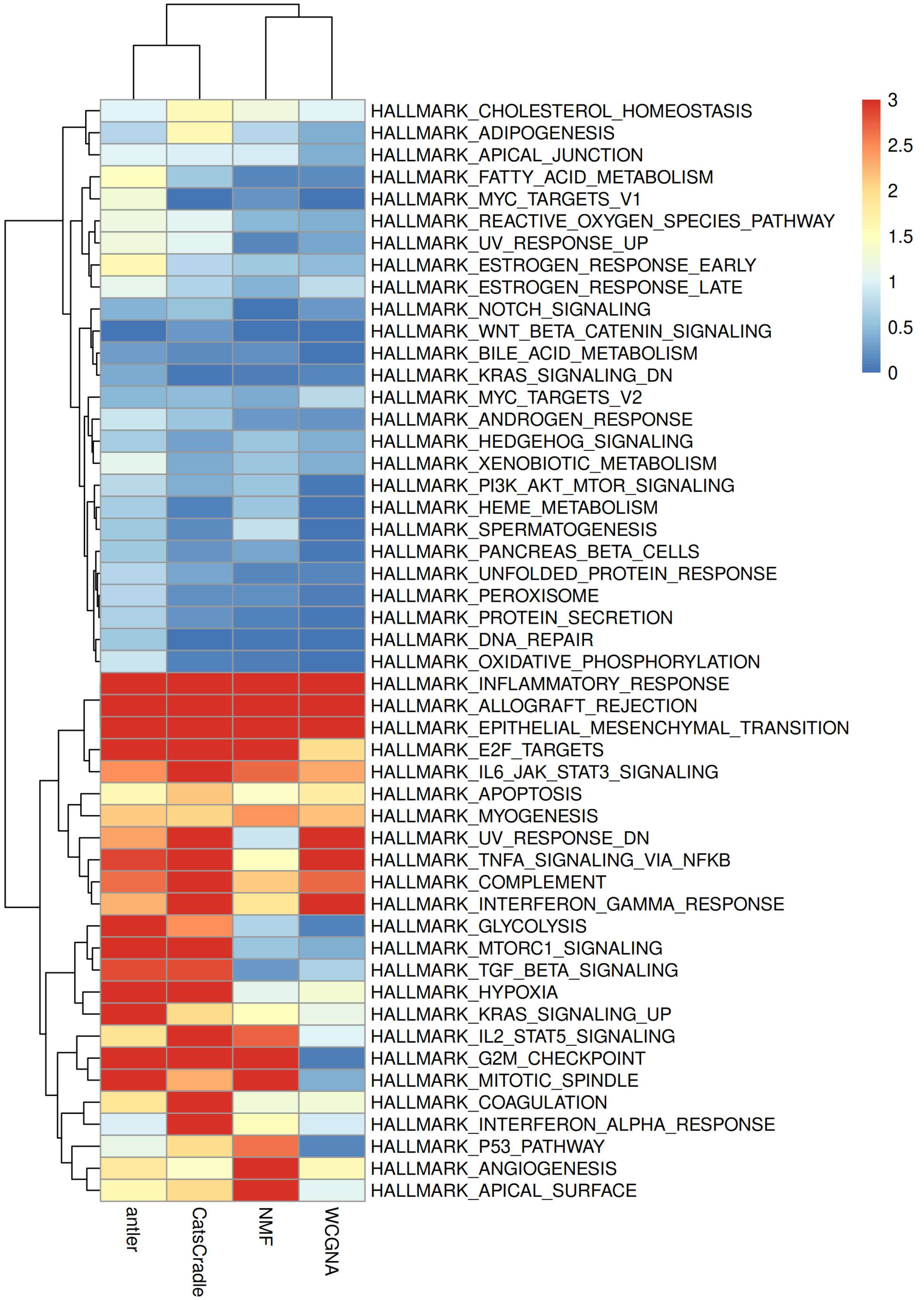

Supplementary Figure 4. Heatmap showing the minimum minus log10 of p-values for overlap between gene modules and Hallmark gene sets for gene modules calculated using the scWCGNA, GeneNMF, Antler and CatsCradle software. We have imposed a minimum p-value of 0.001 for visual clarity.

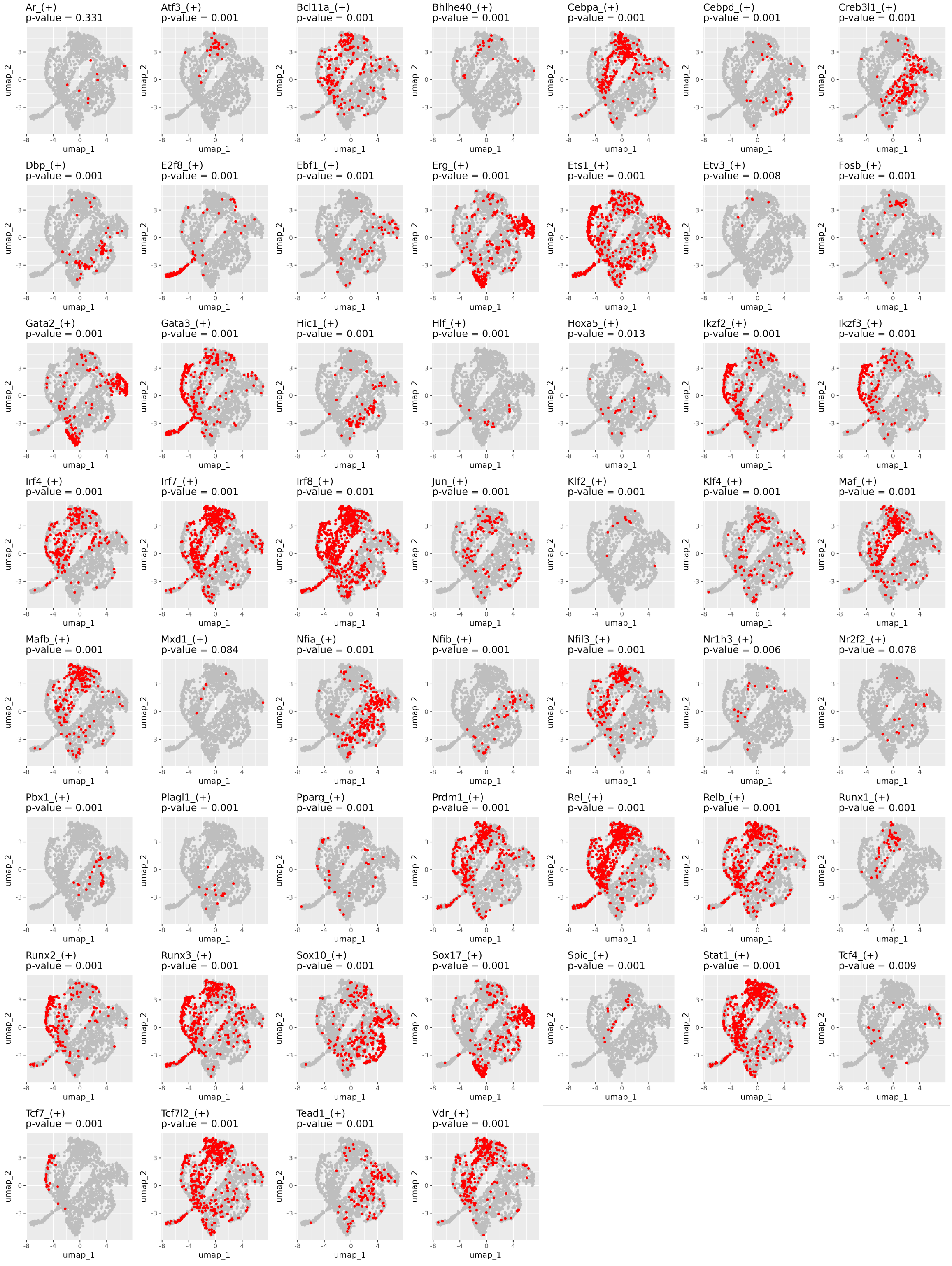

Supplementary Figure 5. For each regulon calculated using pySCENIC we display the overlap of that regulon with the genes of our gene UMAP along with their mcd p-value.

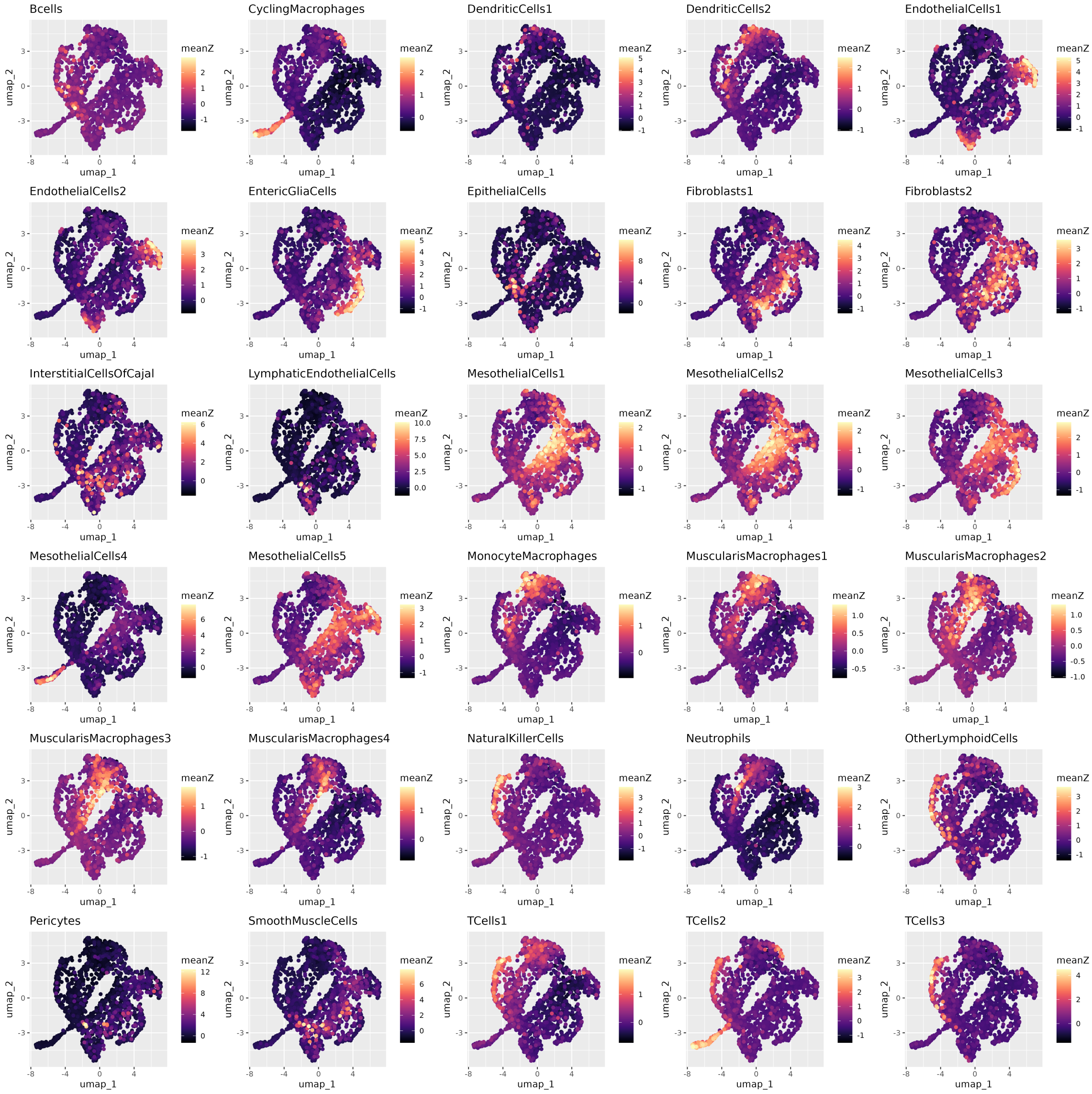

Supplementary Figure 6. For each cell cluster, we display the mean z-score in that cluster of each gene in our gene UMAP.

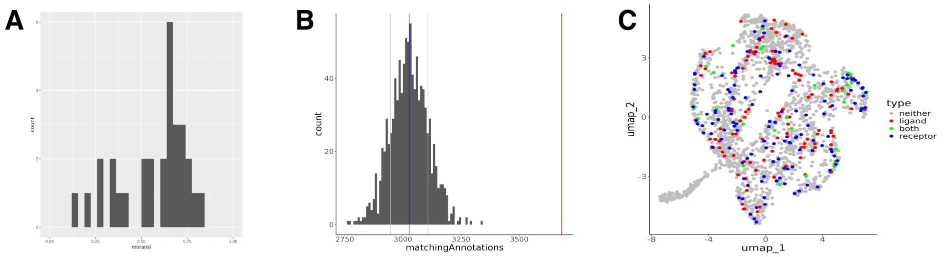

Supplementary Figure 7. **A)** Histogram showing Moran's I for co-localization of z-scores per gene per cell cluster. **B)** Comparison of the total coincidence of gene and neighbouring gene annotations for actual neighbours and randomised neighbours. Grey shows histogram of random coincidences, blue and grey lines show mean and standard deviation of random coincidences, red shows actual coincidences. **C)** Ligands and receptors on gene UMAP. Ligands in red, receptors in blue and genes which are both in green.

Table 1. Table showing p-values for overlap between CatsCradle gene clusters and Hallmark gene sets.

	cluster_0	cluster_1	cluster_2	cluster_3	cluster_4	cluster_5	cluster_6	cluster_7	cluster_8
HALLMARK_ADIPOGENESIS	0.585904730317005	0.999984360412283	0.912660619858443	0.0254916907532096	0.944127314977106	0.932554973044467	0.928219597162865	0.925954935303681	1
HALLMARK_ALLOGRAFT_REJECTION	0.149330866992232	0.926570525942568	0.999674825059323	0.998825464764227	0.94829726492214	0.0115505774957671	1	0.987494179360635	2.2651808418386e-13
HALLMARK_ANDROGEN_RESPONSE	0.879991691721389	0.9998102012773	0.756543768455786	0.569920816725752	1	0.504003620372621	1	0.263326407675457	0.911650793747928
HALLMARK_ANGIOGENESIS	0.0857806910369604	1	0.358266575797139	0.319898429981322	1	0.179427275960224	0.174655249312156	0.0382738758273716	0.499915701162822
HALLMARK_APICAL_JUNCTION	0.993377172631758	0.999870830356879	0.285643820542898	0.102756348588046	0.66174582632105	0.987559424633993	0.986570914709117	0.924637574984138	0.979561382821321
HALLMARK_APICAL_SURFACE	0.958718268660415	0.949859951223618	0.486148850553726	0.761435794640446	1	1	0.609021620902474	0.233375214782849	0.00932332041783182
HALLMARK_APOPTOSIS	0.0373076888668773	0.998763504498005	0.0923862753846423	0.762772894091673	0.975795932407363	0.00725752751540566	0.969024731893836	0.658619194760508	0.0850093429204606
HALLMARK_BILE_ACID_METABOLISM	0.927294135015053	0.995782524347614	0.965683233658289	0.878600177515406	1	1	0.909809769052027	0.68345112782761	0.885985132771177
HALLMARK_CHOLESTEROL_HOMEOSTASIS	0.0274174449217639	0.99400515194797	0.457166009368264	1	0.820823194276037	0.0760738292190059	1	0.0705165082159879	1
HALLMARK_COAGULATION	0.538992432068356	0.999209679913583	0.0339807494494032	0.0755290587360964	1	0.000715777808032286	0.949887636492349	0.948539800089956	0.932916393942028
HALLMARK_COMPLEMENT	0.225959615551485	0.99173627971162	0.628592854316583	0.446622119196337	1	0.000278159353191338	0.925616213266119	1	0.173764135470944
HALLMARK_DNA_REPAIR	0.99398736464592	0.972766861698669	0.99990486054959	1	1	0.962788331865387	1	1	1
HALLMARK_E2F_TARGETS	0.493317872595657	7.90600691509096e-11	0.999995911902774	1	0.944127314977106	0.932554973044467	1	1	1
HALLMARK_EPITHELIAL_MESENCHYMAL_TRANSITION	0.552582617877641	0.99955115875876	2.20631673365261e-17	0.121478024021925	0.99162039894421	0.00139375962596969	0.0286902917684116	0.000315933051228705	0.757570216013273
HALLMARK_ESTROGEN_RESPONSE_EARLY	0.974904007233428	0.980557770614609	0.912660619858443	0.188112592034969	1	0.932554973044467	0.799133871189364	0.237836973524565	0.531277991273675
HALLMARK_ESTROGEN_RESPONSE_LATE	0.725818628687641	0.877568774622741	0.234069123644189	0.649802338543874	1	0.939558781108274	0.935527363294084	0.430185729015415	0.196759605807421
HALLMARK_FATTY_ACID_METABOLISM	0.541654625290324	0.999762867293222	0.986385567250802	0.886560906329069	1	0.249347637020769	0.851203782879596	1	0.953888664386109
HALLMARK_G2M_CHECKPOINT	0.950460926304789	1.1277214964099e-08	0.979992920692483	0.998562202229778	1	1	0.986271205287981	0.985739143954456	1
HALLMARK_GLYCOLYSIS	0.756311743762312	0.999371479544637	0.981510141118215	1	0.835028420507912	0.00346424063435228	0.412557223073661	0.986349711618709	0.979964596299468
HALLMARK_HEDGEHOG_SIGNALING	0.553200706221413	1	0.88898791259898	1	0.560571298688856	1	0.535958167373266	0.532788843530625	0.499915701162822
HALLMARK_HEME_METABOLISM	0.992944725333726	1	0.992798979980388	0.988809662661227	0.829913560305333	0.930056005590883	0.793498720369968	0.985739143954456	0.979150135005458
HALLMARK_HYPOXIA	0.493317872595657	0.992466794105854	0.75308790220562	0.188112592034969	0.665637468891362	5.3226369698925e-09	0.612365093268808	0.405163713921734	0.979964596299468
HALLMARK_IL2_STAT5_SIGNALING	5.68944020014228e-05	0.155474065646496	0.84079397988014	0.886113765525823	0.290734838668607	0.423031566795379	0.124775188621266	0.791385934161196	0.000348643450421301
HALLMARK_IL6_JAK_STAT3_SIGNALING	0.0516072792781311	0.937749374921102	0.779864498466221	0.945589023196985	1	0.000575718655831297	0.851566605700201	1	0.50787742269967
HALLMARK_INFLAMMATORY_RESPONSE	0.000156401762533429	0.918889597812802	0.758559525674914	0.890700185615306	0.475453940215751	0.0275752985915276	0.987151012570358	0.927250761921934	0.182250983447848
HALLMARK_INTERFERON_ALPHA_RESPONSE	3.929597941274e-06	1	0.997852745936266	1	0.899534128288338	0.0623441446769319	1	1	1
HALLMARK_INTERFERON_GAMMA_RESPONSE	4.19664747981072e-05	0.987276918061206	0.995779991993377	1	0.858678631847052	0.000135876340046609	0.989468797117018	1	0.208675131822155
HALLMARK_KRAS_SIGNALING_DN	0.999998759543708	0.999999128697763	0.999948868570198	0.998743522508526	0.990788424303034	0.988370502384745	1	0.92852536965864	0.980747539078645
HALLMARK_KRAS_SIGNALING_UP	0.00969643697290804	0.99791333423326	0.422078883170686	0.0589363987835593	0.0860666171842587	0.637930595473233	0.931965337641108	0.929779069714984	0.748632930981864
HALLMARK_MITOTIC_SPINDLE	0.973516201224803	0.00513864901423475	0.998022973735088	0.451884761287662	0.943036326032414	1	1	0.986047737737134	1
HALLMARK_MTORC1_SIGNALING	0.249893096428342	0.918889597812802	0.999617918846315	0.99870044470313	0.945198580041495	0.000322385481331947	0.929489105409602	0.609334196434132	0.980359930893559
HALLMARK_MYC_TARGETS_V1	0.999889058862292	0.98135398693615	0.999996162514098	1	1	0.933772846305946	1	1	1
HALLMARK_MYC_TARGETS_V2	0.885159627633835	0.330226012392032	1	1	1	1	1	1	0.673319377567646
HALLMARK_MYOGENESIS	0.997463855838272	0.992808726634152	0.0837346790244293	0.107921627844606	0.669499063382238	0.988106143826374	0.247505204628935	0.00877166747555111	0.980359930893559
HALLMARK_NOTCH_SIGNALING	0.874104563612464	0.886297917370282	0.858171097392147	0.272198442352455	1	1	0.494504332158607	1	1
HALLMARK_OXIDATIVE_PHOSPHORYLATION	0.997091099428218	1	0.999574665905666	1	1	0.80593335288961	1	1	1
HALLMARK_P53_PATHWAY	0.483302172981461	0.91339789469473	0.998022973735088	0.957169002881373	0.66174582632105	0.00979659764309883	0.986570914709117	0.924637574984138	0.897723067464538
HALLMARK_PANCREAS_BETA_CELLS	0.98972235620986	1	0.913125112550429	1	1	0.580438613813361	1	1	1
HALLMARK_PEROXISOME	0.685023684031066	0.999129307559999	0.986462142441333	1	0.906289827868424	0.652459979694906	1	0.888197553894871	1
HALLMARK_PI3K_AKT_MTOR_SIGNALING	0.999994407945631	0.993544161133163	1	0.967928411256832	1	0.391222197912509	0.65345265265198	1	0.597950926376131
HALLMARK_PROTEIN_SECRETION	0.982428041841767	1	0.99724832125245	0.817601728326887	1	0.614033778463271	1	1	1
HALLMARK_REACTIVE_OXYGEN_SPECIES_PATHWAY	0.435814353625582	1	0.794788213395507	0.467167741505618	0.673909897000002	0.0866270306384667	1	1	1
HALLMARK_SPERMATOGENESIS	0.999999831477918	0.667697073152882	1	0.988148203437171	0.813625446517072	1	0.945318857952457	0.943891565961657	1
HALLMARK_TGF_BETA_SIGNALING	0.362431171534068	0.974693212038521	0.837302329565455	0.00149169188223094	0.70931279536008	0.690896566424487	1	1	1
HALLMARK_TNFA_SIGNALING_VIA_NFKB	3.7485522988919e-14	0.92152262278754	0.98291993276177	0.63078035072467	0.990788424303034	0.00117603556241595	0.930737581268802	1	0.185117005252977
HALLMARK_UNFOLDED_PROTEIN_RESPONSE	0.931417653979838	0.996032632463817	1	1	0.925717035277902	0.437380066719441	0.911746786069372	1	1
HALLMARK_UV_RESPONSE_DN	0.865054274918881	0.999423215379152	0.000726306924924322	0.0399552393641036	0.407926476888069	0.371478351043659	0.359287593207336	0.180987075576911	0.939210380489742
HALLMARK_UV_RESPONSE_UP	0.0855167647503381	0.954764671082063	0.986986933308792	1	0.700896124552698	0.25339882894425	0.431288749013097	1	0.954791261795503
HALLMARK_WNT_BETA_CATENIN_SIGNALING	0.991832718444994	0.942521463738673	1	1	0.617056147272135	1	1	1	0.554665540460346
HALLMARK_XENOBIOTIC_METABOLISM	0.843910738966867	0.993449004294668	0.920376572423639	0.961215076286074	0.991003796521322	0.816691910360218	0.425175628629733	0.929779069714984	0.748632930981864

Table 2. Table showing p-values for overlap between scWCNA gene clusters and Hallmark gene sets.

	cluster_1_brown	cluster_2_pink	cluster_3_turquoise	cluster_4_yellow
HALLMARK_ADIPOGENESIS	0.403753367588022	1	0.955061866189796	0.996916082017909
HALLMARK_ALLOGRAFT_REJECTION	0.999998338339819	1	0.00317305799750902	9.54450399108124e-06
HALLMARK_ANDROGEN_RESPONSE	0.576295094679578	1	0.917893365777407	1
HALLMARK_ANGIOGENESIS	0.0261787878682877	0.219172947686025	0.690444563321832	0.75837014053899
HALLMARK_APICAL_JUNCTION	0.566956709733343	0.396474803554068	0.97875576665417	0.985082730709654
HALLMARK_APICAL_SURFACE	0.64070148609955	0.261109179934487	0.791238728377947	0.0880224142481744
HALLMARK_APOPTOSIS	0.123539907193794	0.0959883849025863	0.0164351664616809	0.751379962431982
HALLMARK_BILE_ACID_METABOLISM	0.942909072642699	1	0.995613358698013	0.934780697202687
HALLMARK_CHOLESTEROL_HOMEOSTASIS	0.438921235814702	0.0922776767694054	0.112946062513236	1
HALLMARK_COAGULATION	0.0497227957972714	0.245081610490415	0.117844309662173	0.973542174218882
HALLMARK_COMPLEMENT	0.854140651918996	0.74883578452019	0.00213002340703821	0.886833236158884
HALLMARK_DNA_REPAIR	0.999999948318703	1	0.990397056529457	1
HALLMARK_E2F_TARGETS	0.999999912303216	0.752382715589099	0.0101865469749592	0.95457030059663
HALLMARK_EPITHELIAL_MESENCHYMAL_TRANSITION	2.62717399004829e-21	0.164636175193298	0.184830168029885	0.689072174577628
HALLMARK_ESTROGEN_RESPONSE_EARLY	0.322900955920478	0.398916684601317	0.992059176881502	0.95457030059663
HALLMARK_ESTROGEN_RESPONSE_LATE	0.305276022791133	0.164636175193298	0.589836446348148	0.905960213347097
HALLMARK_FATTY_ACID_METABOLISM	0.930673638757475	0.66657520157364	0.980294641624247	0.998176202162614
HALLMARK_G2M_CHECKPOINT	0.999572943081836	1	0.840918567625645	0.996684609496349
HALLMARK_GLYCOLYSIS	0.943028072571396	1	0.764068405822759	0.95457030059663
HALLMARK_HEDGEHOG_SIGNALING	0.991094644308059	1	0.912193745357128	0.407129208818619
HALLMARK_HEME_METABOLISM	0.997380244555118	1	0.991296003577569	0.999641706871599
HALLMARK_HYPOXIA	0.250365677200915	0.045959871447187	0.154887046012837	0.985547387005792
HALLMARK_IL2_STAT5_SIGNALING	0.313108693293337	0.153097049250048	0.095922842711622	0.237577658808114
HALLMARK_IL6_JAK_STAT3_SIGNALING	0.53651435439089	1	0.00464951591871312	0.864176140778161
HALLMARK_INFLAMMATORY_RESPONSE	0.91421434081513	1	1.46701898781761e-08	0.795428203216127
HALLMARK_INTERFERON_ALPHA_RESPONSE	0.998920885051075	0.499102209436475	0.114415848423875	0.981117076226318
HALLMARK_INTERFERON_GAMMA_RESPONSE	0.99995011059258	0.423096285419215	3.04842516288213e-05	0.989493953541655
HALLMARK_KRAS_SIGNALING_DN	0.999999999939016	0.755880892186705	0.999985204427918	0.999695728683179
HALLMARK_KRAS_SIGNALING_UP	0.521982865441393	0.406216995713618	0.0683014469089094	0.997233918324124
HALLMARK_MITOTIC_SPINDLE	0.940068586087117	0.396474803554068	0.991685855502649	0.985082730709654
HALLMARK_MTORC1_SIGNALING	0.995375400376142	0.40135438136675	0.553086091504893	0.985998214566617
HALLMARK_MYC_TARGETS_V1	0.999999999930475	1	0.999984207672779	1
HALLMARK_MYC_TARGETS_V2	1	1	0.173920377975167	1
HALLMARK_MYOGENESIS	0.00651453555123606	0.40135438136675	0.997548446694956	0.985998214566617
HALLMARK_NOTCH_SIGNALING	0.56335029892882	1	1	1
HALLMARK_OXIDATIVE_PHOSPHORYLATION	0.999863580164917	1	0.999982010103722	1
HALLMARK_P53_PATHWAY	0.905919598035327	0.75061538543416	0.758357471731338	0.996802395067787
HALLMARK_PANCREAS_BETA_CELLS	1	1	0.933059562979687	1
HALLMARK_PEROXISOME	0.827318281448854	1	0.992449287831525	1
HALLMARK_PI3K_AKT_MTOR_SIGNALING	0.999984783854147	1	0.918675939111826	0.918145868866474
HALLMARK_PROTEIN_SECRETION	0.994084898121626	1	0.88059783168539	1
HALLMARK_REACTIVE_OXYGEN_SPECIES_PATHWAY	0.566083078195821	1	0.397060220060386	1
HALLMARK_SPERMATOGENESIS	0.999976143766931	1	0.998933720343712	0.995377542739245
HALLMARK_TGF_BETA_SIGNALING	0.209861462702767	0.310456520105307	0.875850677438296	1
HALLMARK_TNFA_SIGNALING_VIA_NFKB	0.822747468890044	1	2.28069713446465e-11	0.986435599528451
HALLMARK_UNFOLDED_PROTEIN_RESPONSE	0.999994389677562	1	0.75475400158094	1
HALLMARK_UV_RESPONSE_DN	5.95530005741794e-06	0.25783264783831	0.963103351102916	1
HALLMARK_UV_RESPONSE_UP	0.934354040495365	0.668919693694844	0.463369812932809	0.998248641111306
HALLMARK_WNT_BETA_CATENIN_SIGNALING	1	1	1	1
HALLMARK_XENOBIOTIC_METABOLISM	0.92190061661097	0.406216995713618	0.780641592632492	0.986859918264172

Table 3. Table showing p-values for overlap between geneNMF gene clusters and Hallmark gene sets.

	cluster_MP1	cluster_MP10	cluster_MP2	cluster_MP3	cluster_MP4	cluster_MP5	cluster_MP6	cluster_MP7	cluster_MP8	cluster_MP9
HALLMARK_ADIPOGENESIS	0.178312801645526	1	1	0.507776169349459	1	1	0.801401427623647	1	1	0.823932084892015
HALLMARK_ALLOGRAFT_REJECTION	0.00315902361957175	1	0.0306081847947373	0.979197249571672	0.0302034405372455	2.775863426462e-09	1	1	1	1
HALLMARK_ANDROGEN_RESPONSE	0.561892109624376	1	1	0.676858486228257	1	1	0.847490473624131	1	1	1
HALLMARK_ANGIOGENESIS	0.419484811626987	1	1	0.000463022802252133	0.0650775895719808	1	1	1	1	1
HALLMARK_APICAL_JUNCTION	1	1	0.828944811168815	0.503801773677378	0.679501954471199	0.85291921915614	0.568184021739578	1	0.109988856843483	0.822367036757722
HALLMARK_APICAL_SURFACE	1	1	1	0.560751147025308	0.403394078360932	0.000715363586087366	0.137029774576308	1	1	0.313759763141209
HALLMARK_APOPTOSIS	0.434223940471309	0.799320400401069	0.759111922585954	0.345904837632144	0.0383274297930424	0.0637713501885353	0.426276510566763	0.214470816733499	1	0.751672256277943
HALLMARK_BILE_ACID_METABOLISM	0.818059885169599	1	1	0.878468537429705	1	0.656960830273912	1	1	1	0.618850797869013
HALLMARK_CHOLESTEROL_HOMEOSTASIS	0.309192033004844	1	1	1	0.0559832515926038	1	0.304055780117937	1	1	0.474639805936824
HALLMARK_COAGULATION	0.056073791575114	1	0.706640443532449	0.113124205050256	0.483812140799126	1	0.0536723390931329	1	0.728828257537217	1
HALLMARK_COMPLEMENT	0.0277374588169013	1	1	0.714497659372435	0.00763112822796486	0.114545649257354	0.167194796585458	1	0.845782369500857	1
HALLMARK_DNA_REPAIR	1	1	1	1	1	1	0.895857730153574	1	1	1
HALLMARK_E2F_TARGETS	1	1.60505552348345e-18	1	1	1	1	1	1	1	1
HALLMARK_EPITHELIAL_MESENCHYMAL_TRANSITION	0.957679668033611	1	1	6.91106598399152e-21	0.00940447897854974	0.862691750692411	0.591981963988925	1	1	0.253904436830378
HALLMARK_ESTROGEN_RESPONSE_EARLY	1	1	0.250129639972422	0.97752329316207	0.682198339443069	0.854355940056932	0.801401427623647	0.25991144258312	1	0.510922458033375
HALLMARK_ESTROGEN_RESPONSE_LATE	1	0.604470492757614	0.839442909456537	0.899388892915783	0.697997935431393	0.862691750692411	0.362012004284157	1	1	0.833042994469704
HALLMARK_FATTY_ACID_METABOLISM	0.910586822200523	1	0.752538790875425	1	1	0.780394951680361	0.90786060026067	1	1	1
HALLMARK_G2M_CHECKPOINT	1	9.63120207948035e-29	1	0.976636984781442	1	1	0.95019085528458	1	1	1
HALLMARK_GLYCOLYSIS	1	0.588120659151376	1	0.889740802094192	0.202179107822845	1	0.801401427623647	1	1	1
HALLMARK_HEDGEHOG_SIGNALING	1	1	1	1	1	0.289266808012798	1	1	1	0.264965854527702
HALLMARK_HEME_METABOLISM	1	1	1	1	0.905422649539365	0.851468606156133	0.796782950891763	0.257638401013474	1	1
HALLMARK_HYPOXIA	1	1	1	0.507776169349459	0.0817227872938791	1	0.801401427623647	1	1	0.823932084892015
HALLMARK_IL2_STAT5_SIGNALING	0.952781668583316	1	0.0278809002378852	0.97708438070673	0.00195816398786379	0.287966038098838	0.338251895100114	1	0.847258984116896	0.508214767530556
HALLMARK_IL6_JAK_STAT3_SIGNALING	0.00200186973309426	1	1	0.81196660019859	0.019387418210766	1	0.736680923278691	0.124133982162927	1	1
HALLMARK_INFLAMMATORY_RESPONSE	0.000172379728065108	1	1	0.97795388141567	0.000461188956965104	0.293154040302984	0.575067193654089	0.00245111756560323	0.558916645514912	1
HALLMARK_INTERFERON_ALPHA_RESPONSE	0.781238600331403	1	0.584814075620316	0.847353924848428	0.0283411151715996	1	1	1	1	1
HALLMARK_INTERFERON_GAMMA_RESPONSE	0.0125949078179294	1	0.107406837748007	0.905382455832928	0.0341409646729363	0.867987187444155	0.958622331359051	0.0358581820375981	1	1
HALLMARK_KRAS_SIGNALING_DN	0.954945831430169	1	1	1	1	1	0.953174530914189	1	0.851606186825785	0.827021819523545
HALLMARK_KRAS_SIGNALING_UP	0.0312148492421545	1	1	0.51961620837288	0.421997767589561	0.575232132468079	0.35182421246917	0.263309586453487	0.116090254569812	0.518989383151904
HALLMARK_MITOTIC_SPINDLE	0.952781668583316	9.13043304613881e-15	1	1	1	1	0.950953921461759	1	0.847258984116896	1
HALLMARK_MTORC1_SIGNALING	0.95423555746089	0.313675746699096	0.832010955730067	1	1	1	1	0.261045677920157	1	1
HALLMARK_MYC_TARGETS_V1	1	0.59087889947753	1	1	1	1	1	1	1	1
HALLMARK_MYC_TARGETS_V2	1	0.436159129604036	1	1	1	1	1	1	1	1
HALLMARK_MYOGENESIS	0.95423555746089	1	0.25247777454117	0.00370744965162846	1	1	0.345035550961574	1	0.558916645514912	0.0895186717324451
HALLMARK_NOTCH_SIGNALING	1	1	1	1	1	1	1	1	1	1
HALLMARK_OXIDATIVE_PHOSPHORYLATION	1	1	0.828944811168815	1	1	1	1	1	1	1
HALLMARK_P53_PATHWAY	0.952781668583316	1	0.519789413847448	1	0.90656366315082	1	0.799103752202952	0.00238167800496135	0.847258984116896	1
HALLMARK_PANCREAS_BETA_CELLS	0.453648053392001	1	1	1	1	1	0.449524642474205	1	1	1
HALLMARK_PEROXISOME	1	0.640070212392227	1	1	1	1	1	1	1	1
HALLMARK_PI3K_AKT_MTOR_SIGNALING	1	0.647210697123867	1	1	1	0.259716260865492	1	1	1	1
HALLMARK_PROTEIN_SECRETION	0.767399301544255	1	1	1	1	1	1	1	1	1
HALLMARK_REACTIVE_OXYGEN_SPECIES_PATHWAY	1	1	0.34862528249	1	0.437554101067092	1	1	1	1	1
HALLMARK_SPERMATOGENESIS	1	0.144707514197958	1	1	1	1	1	1	0.718315879161582	0.68813120643639
HALLMARK_TGF_BETA_SIGNALING	0.558304443028136	1	1	1	1	1	1	1	1	1
HALLMARK_TNFA_SIGNALING_VIA_NFKB	0.183395473103679	1	0.833523790579787	0.978376301775592	0.0844579528515799	1	1	0.0333701558078008	0.851606186825785	1
HALLMARK_UNFOLDED_PROTEIN_RESPONSE	1	1	1	1	0.736864609463119	1	1	1	1	1
HALLMARK_UV_RESPONSE_DN	1	1	1	0.126215963437486	1	1	0.357879592467144	1	1	1
HALLMARK_UV_RESPONSE_UP	0.91196319380861	1	0.754749105742178	1	1	0.782523392855637	0.90926142731866	1	1	1
HALLMARK_WNT_BETA_CATENIN_SIGNALING	1	1	1	1	1	1	1	1	1	1
HALLMARK_XENOBIOTIC_METABOLISM	0.590400779396817	1	1	0.894666316315063	0.421997767589561	0.858584048225681	0.808157363335286	0.263309586453487	1	0.82854673257489

Table 4. Table showing p-values for overlap between Antler gene clusters and Hallmark gene sets.

	cluster_1	cluster_2	cluster_3	cluster_4	cluster_5	cluster_6	cluster_7	cluster_8	cluster_9
HALLMARK_ADIPOGENESIS	1	1	1	1	0.180453339438389	1	1	1	1
HALLMARK_ALLOGRAFT_REJECTION	1	3.63589248019109e-06	4.83405184364976e-11	1	0.186076457612582	0.205703338856887	0.0519910134485846	1	0.00373067241730708
HALLMARK_ANDROGEN_RESPONSE	1	1	1	1	0.387928745217952	1	0.208261878537843	1	1
HALLMARK_ANGIOGENESIS	1	1	1	1	0.130822931102576	1	1	1	1
HALLMARK_APICAL_JUNCTION	1	1	0.768714830784088	1	1	1	1	1	1
HALLMARK_APICAL_SURFACE	1	0.100650653830562	0.0394774307006307	1	1	1	1	1	1
HALLMARK_APOPTOSIS	0.683324121401472	1	0.0275103862635457	1	1	0.165576581079524	0.26051677934825	0.557879154280807	1
HALLMARK_BILE_ACID_METABOLISM	1	1	0.558353670035559	1	1	1	1	1	1
HALLMARK_CHOLESTEROL_HOMEOSTASIS	1	1	1	1	1	1	0.130171616592632	1	1
HALLMARK_COAGULATION	1	1	1	1	0.421090777886156	1	0.0256768597155044	0.151467037228185	1
HALLMARK_COMPLEMENT	1	0.38390000899177	0.0524875614925185	1	1	1	0.049261099703638	0.01568387376351	1
HALLMARK_DNA_REPAIR	1	1	1	1	1	1	1	1	1
HALLMARK_E2F_TARGETS	3.22867619808948e-15	1	1	1	1	1	1	1	1
HALLMARK_EPITHELIAL_MESENCHYMAL_TRANSITION	1	1	0.780547447977417	1	1	1	0.321461065171763	1	1
HALLMARK_ESTROGEN_RESPONSE_EARLY	1	0.386964005757212	1	0.0724456589463476	1	1	1	1	1
HALLMARK_ESTROGEN_RESPONSE_LATE	0.426778788126482	1	1	0.0745769463599786	1	1	1	1	1
HALLMARK_FATTY_ACID_METABOLISM	1	1	1	1	0.463368515225155	1	1	1	1
HALLMARK_G2M_CHECKPOINT	9.95134675864432e-23	1	1	1	1	1	1	1	1
HALLMARK_GLYCOLYSIS	1	1	1	1	1	1	1	1	1
HALLMARK_HEDGEHOG_SIGNALING	1	1	0.229582894824547	1	1	1	1	1	1
HALLMARK_HEME_METABOLISM	1	1	1	1	1	1	0.310968735264155	1	1
HALLMARK_HYPOXIA	1	1	0.770442492457986	0.0724456589463476	1	1	1	1	1
HALLMARK_IL2_STAT5_SIGNALING	1	0.0802153332247867	0.768714830784088	1	1	1	1	0.636676562876252	1
HALLMARK_IL6_JAK_STAT3_SIGNALING	1	1	1	1	0.294144704126847	1	0.0110137168904812	0.361173973158867	1
HALLMARK_INFLAMMATORY_RESPONSE	1	0.388490723750537	0.174630893731765	1	0.548457649405905	1	0.0506189855918446	0.000371984499702583	1
HALLMARK_INTERFERON_ALPHA_RESPONSE	1	1	1	1	1	0.105765406446651	1	1	1
HALLMARK_INTERFERON_GAMMA_RESPONSE	1	1	0.0163944941907248	1	1	0.0202169173236502	0.00562274739765965	1	1
HALLMARK_KRAS_SIGNALING_DN	1	1	1	1	0.550274547156906	1	1	1	1
HALLMARK_KRAS_SIGNALING_UP	1	1	0.432440177135553	1	0.552084480945658	1	1	0.644136187835664	0.290942283044385
HALLMARK_MITOTIC_SPINDLE	1.29327229069874e-12	1	1	1	1	1	1	1	1
HALLMARK_MTORC1_SIGNALING	0.165422649392672	1	1	1	1	1	0.314920348328741	0.640425002804731	0.288490212055052
HALLMARK_MYC_TARGETS_V1	0.414461699809754	1	1	1	1	1	1	1	1
HALLMARK_MYC_TARGETS_V2	0.336511006114128	1	1	1	1	1	1	1	1
HALLMARK_MYOGENESIS	1	1	1	1	0.548457649405905	1	1	1	0.288490212055052
HALLMARK_NOTCH_SIGNALING	1	1	1	1	1	1	1	1	1
HALLMARK_OXIDATIVE_PHOSPHORYLATION	1	1	1	1	1	1	1	1	1
HALLMARK_P53_PATHWAY	1	1	1	0.0720902060469212	1	0.201121751018339	1	1	1
HALLMARK_PANCREAS_BETA_CELLS	1	1	1	1	1	1	1	1	1
HALLMARK_PEROXISOME	0.518868403825639	1	1	1	1	1	1	1	1
HALLMARK_PI3K_AKT_MTOR_SIGNALING	1	1	0.174110623086245	1	1	1	1	1	1
HALLMARK_PROTEIN_SECRETION	1	1	1	1	1	1	1	0.383493420321831	1
HALLMARK_REACTIVE_OXYGEN_SPECIES_PATHWAY	1	1	1	1	1	1	1	1	0.0785400831115594
HALLMARK_SPERMATOGENESIS	0.24419287508447	1	1	1	1	1	1	1	1
HALLMARK_TGF_BETA_SIGNALING	1	1	1	1	1	1	1	1	1
HALLMARK_TNFA_SIGNALING_VIA_NFKB	1	1	0.773860174426796	0.00138238492552853	1	1	0.316233012922861	0.642285229845212	1
HALLMARK_UNFOLDED_PROTEIN_RESPONSE	1	1	1	1	1	1	0.190119124128483	1	1
HALLMARK_UV_RESPONSE_DN	1	1	1	1	1	1	0.23624076772219	1	1
HALLMARK_UV_RESPONSE_UP	1	1	1	0.0578162526412124	1	1	1	1	1
HALLMARK_WNT_BETA_CATENIN_SIGNALING	1	1	1	1	1	1	1	1	1
HALLMARK_XENOBIOTIC_METABOLISM	1	1	1	1	0.552084480945658	1	1	1	1

	cluster_10	cluster_11	cluster_12	cluster_13	cluster_14	cluster_15	cluster_16	cluster_17	cluster_18
HALLMARK_ADIPOGENESIS	1	1	0.231517482966128	1	0.384055208093077	1	0.947875129936116	0.207571257008564	1
HALLMARK_ALLOGRAFT_REJECTION	0.0131307725669396	1	0.0261283271762123	1	1	1	1	1	0.908588222070811
HALLMARK_ANDROGEN_RESPONSE	1	1	1	0.226555273211183	0.588312988033474	0.278957165355718	0.840093906146725	1	1
HALLMARK_ANGIOGENESIS	0.224394900850302	1	0.0456029408703518	1	1	1	0.0145587078525744	1	1
HALLMARK_APICAL_JUNCTION	0.759834694053629	1	1	1	0.0911140585339814	1	0.787412551550209	1	0.670927420400351
HALLMARK_APICAL_SURFACE	1	1	1	1	1	1	0.0248864372031705	0.171270100188254	1
HALLMARK_APOPTOSIS	0.0252558985639262	1	0.190390428594522	0.0404487598936632	0.724823997892569	1	0.200902869824917	0.148034692807207	0.55454724237008
HALLMARK_BILE_ACID_METABOLISM	1	1	1	1	0.528262422018077	1	0.80611675780808	1	1
HALLMARK_CHOLESTEROL_HOMEOSTASIS	0.0965611596795153	1	1	0.142230235586264	0.329660283342724	1	0.665367722996228	1	0.580569740841365
HALLMARK_COAGULATION	0.0155608955556037	1	0.0126016482558649	0.248775849000915	0.0213033207878696	1	0.323354578624946	1	0.212013758739935
HALLMARK_COMPLEMENT	0.0022889273202449	1	0.0247024120876154	1	0.958768244474899	0.406461096141692	0.785034910264055	1	0.396297447691388
HALLMARK_DNA_REPAIR	1	0.244889456960644	1	1	1	1	1	0.476300787355775	1
HALLMARK_E2F_TARGETS	0.7615826890021	1	1	1	1	1	1	1	1
HALLMARK_EPITHELIAL_MESENCHYMAL_TRANSITION	0.0546150183931876	1	0.0266112644733584	0.347298222394428	0.0418598207572693	0.0969558999977446	3.71312323830965e-10	1	1
HALLMARK_ESTROGEN_RESPONSE_EARLY	0.7615826890021	1	0.0251738842228563	1	0.960099728921969	0.092180653615312	0.947875129936116	0.579603530713463	0.1945310874718
HALLMARK_ESTROGEN_RESPONSE_LATE	0.426778788126482	1	0.237682927124398	1	0.63335322319169	0.419080721816731	0.952379334865039	0.590600963816157	0.0854238150241821
HALLMARK_FATTY_ACID_METABOLISM	1	0.0325886140008184	1	0.278004273596866	1	1	1	0.494320452584257	0.841943773607176
HALLMARK_G2M_CHECKPOINT	1	1	1	1	1	1	0.547378436219163	1	1
HALLMARK_GLYCOLYSIS	0.0499404465095262	9.17690341368915e-09	1	1	0.960099728921969	1	0.554280975316261	1	0.904111876491495
HALLMARK_HEDGEHOG_SIGNALING	1	1	1	1	1	1	1	1	1
HALLMARK_HEME_METABOLISM	1	1	0.229452859958952	1	1	1	1	0.575875336549196	0.901793518649596
HALLMARK_HYPOXIA	0.0121249834173032	4.35091168848814e-07	0.231517482966128	0.338980447001114	0.204018131984455	1	0.789767415956613	0.579603530713463	0.904111876491495
HALLMARK_IL2_STAT5_SIGNALING	0.0118820662724732	1	0.230485764972412	0.337584820305723	0.825882291681438	1	0.550837243577917	1	0.670927420400351
HALLMARK_IL6_JAK_STAT3_SIGNALING	0.00327475569456286	1	0.109492345125455	1	0.408876529350211	1	1	1	0.643849585912295
HALLMARK_INFLAMMATORY_RESPONSE	0.00246972494808575	1	0.232548015077093	1	0.960749445979753	1	0.948654007304887	1	0.40565776578176
HALLMARK_INTERFERON_ALPHA_RESPONSE	0.15481058300418	1	0.122282484273643	0.185384149951571	1	1	0.768476036406005	1	1
HALLMARK_INTERFERON_GAMMA_RESPONSE	0.014744154890862	1	1	0.35279042357991	1	0.425296674527871	0.81209707974913	1	0.914919876775721
HALLMARK_KRAS_SIGNALING_DN	1	1	1	1	1	1	1	1	0.906376367111685
HALLMARK_KRAS_SIGNALING_UP	0.168863869629156	1	1	1	1	0.09455883771032	0.950177455248162	0.585137102316024	0.201611468955955
HALLMARK_MITOTIC_SPINDLE	1	1	1	1	0.0357167050738411	0.408052353256594	1	0.206004842992137	0.902959509044794
HALLMARK_MTORC1_SIGNALING	0.0507032720014696	1.4454704695162e-05	1	0.340373400082146	0.61656942477121	1	0.948654007304887	1	0.676331659727996
HALLMARK_MYC_TARGETS_V1	1	0.0506189855918446	1	1	1	1	1	1	1
HALLMARK_MYC_TARGETS_V2	0.336511006114128	1	1	1	1	1	1	1	1
HALLMARK_MYOGENESIS	0.763318293948061	1	1	1	0.0945059243101062	0.411222990169279	0.00786771386164827	1	0.676331659727996
HALLMARK_NOTCH_SIGNALING	1	1	1	1	1	1	0.371992938070347	1	1
HALLMARK_OXIDATIVE_PHOSPHORYLATION	1	0.312288212982914	1	1	0.959439385650623	1	0.947084591366219	1	1
HALLMARK_P53_PATHWAY	0.162003870278823	1	1	0.337584820305723	0.380454466570905	1	1	0.577743369344939	0.670927420400351
HALLMARK_PANCREAS_BETA_CELLS	0.24606687195791	1	1	1	1	1	1	1	0.369899043719812
HALLMARK_PEROXISOME	1	1	1	0.190427723519232	1	1	1	1	1
HALLMARK_PI3K_AKT_MTOR_SIGNALING	0.525721657431729	1	1	1	1	1	1	1	1
HALLMARK_PROTEIN_SECRETION	1	1	1	1	0.446834185646164	1	1	1	1
HALLMARK_REACTIVE_OXYGEN_SPECIES_PATHWAY	1	0.0868844808065387	0.0616265808842565	0.0951548431800101	0.540684287356599	1	0.510062086760829	1	1
HALLMARK_SPERMATOGENESIS	1	1	1	1	1	1	1	1	1
HALLMARK_TGF_BETA_SIGNALING	1	1	1	1	0.00145770968120723	1	1	1	1
HALLMARK_TNFA_SIGNALING_VIA_NFKB	0.051472610612064	1	0.233577362441427	1	0.832258218649629	1	1	1	0.906376367111685
HALLMARK_UNFOLDED_PROTEIN_RESPONSE	1	1	1	1	1	1	1	1	1
HALLMARK_UV_RESPONSE_DN	1	1	1	0.25657003379391	0.0747237847864846	0.0517649501210784	0.00441666117310447	0.123484402025642	0.494104631631913
HALLMARK_UV_RESPONSE_UP	0.678698955946845	1	0.188231470676143	1	1	1	0.90360826715715	1	0.547698468828743
HALLMARK_WNT_BETA_CATENIN_SIGNALING	1	1	1	1	1	1	1	1	1
HALLMARK_XENOBIOTIC_METABOLISM	0.419403280496089	1	1	1	1	1	1	1	0.681665294230648

	cluster_19	cluster_20	cluster_21	cluster_22	cluster_23	cluster_24	cluster_25	cluster_26	cluster_27
HALLMARK_ADIPOGENESIS	1	0.865005553430639	0.221288956187232	0.202039826002804	0.409639648229666	1	1	0.700807883819888	1
HALLMARK_ALLOGRAFT_REJECTION	1	0.870406680529618	1	1	0.415949388583352	1	1	1	1
HALLMARK_ANDROGEN_RESPONSE	1	0.346707265609022	1	0.130696007957441	1	1	1	1	1
HALLMARK_ANGIOGENESIS	1	1	1	1	0.0891814467036848	0.181650114108218	1	1	0.249999550567886
HALLMARK_APICAL_JUNCTION	1	0.585349131886913	0.219642832285792	1	0.408052353256594	0.675437208420899	1	0.698962798501376	0.801085314684435
HALLMARK_APICAL_SURFACE	1	1	1	1	1	1	1	1	0.296630492313134
HALLMARK_APOPTOSIS	0.324603418903939	0.799320400401069	0.515811817840372	1	0.0631636104641276	0.223499405585117	1	1	0.727955415237887
HALLMARK_BILE_ACID_METABOLISM	1	0.671124792595768	1	1	1	1	1	1	0.594000822314966
HALLMARK_CHOLESTEROL_HOMEOSTASIS	1	1	1	1	1	1	1	1	0.452024007925644
HALLMARK_COAGULATION	0.286893014163103	1	0.125024068949556	1	0.305225152323142	0.178884983994571	1	1	1
HALLMARK_COMPLEMENT	0.079515867560832	0.86222277721516	0.591435218587969	1	0.406461096141692	1	1	0.697106692442039	1
HALLMARK_DNA_REPAIR	1	1	1	1	1	1	1	1	1
HALLMARK_E2F_TARGETS	1	0.865005553430639	1	1	1	1	1	1	1
HALLMARK_EPITHELIAL_MESENCHYMAL_TRANSITION	0.0851683642951632	0.327227219447791	1.19804886210036e-11	0.207529832459964	0.0144431500796749	0.00495379680752781	0.0210146218256393	1	0.0224879914942575
HALLMARK_ESTROGEN_RESPONSE_EARLY	0.386964005757212	0.865005553430639	1	0.202039826002804	1	1	1	1	0.4750724891251
HALLMARK_ESTROGEN_RESPONSE_LATE	0.396071805972518	0.604470492757614	1	0.207529832459964	1	1	1	1	0.812279890494726
HALLMARK_FATTY_ACID_METABOLISM	1	0.793131100130945	1	1	1	0.589326909811749	1	1	1
HALLMARK_G2M_CHECKPOINT	1	0.86222277721516	1	1	1	1	1	1	1
HALLMARK_GLYCOLYSIS	1	0.588120659151376	0.595182425258082	1	1	1	1	1	1
HALLMARK_HEDGEHOG_SIGNALING	1	1	1	1	1	1	1	1	0.249999550567886
HALLMARK_HEME_METABOLISM	1	0.582564329264656	1	1	1	1	1	1	1
HALLMARK_HYPOXIA	1	0.31096793270508	0.0566765660432078	0.202039826002804	1	1	1	1	0.802723570089256
HALLMARK_IL2_STAT5_SIGNALING	0.0802153332247867	0.585349131886913	1	1	0.408052353256594	0.675437208420899	0.450834431381354	0.698962798501376	1
HALLMARK_IL6_JAK_STAT3_SIGNALING	1	0.585959797238883	1	1	1	1	1	1	1
HALLMARK_INFLAMMATORY_RESPONSE	1	0.132252209100055	1	1	1	1	1	0.702642011735169	1
HALLMARK_INTERFERON_ALPHA_RESPONSE	1	0.629092857033935	1	1	1	1	1	1	1
HALLMARK_INTERFERON_GAMMA_RESPONSE	0.402074104131653	0.878113098083003	1	1	1	1	1	1	1
HALLMARK_KRAS_SIGNALING_DN	1	0.867733163535982	1	1	0.41280238816461	1	1	1	0.805960640354553
HALLMARK_KRAS_SIGNALING_UP	0.0112122205157089	0.596355480288803	1	0.000952345755089715	0.414377851285671	1	0.457537241799894	0.70627764694857	0.482958364026391
HALLMARK_MITOTIC_SPINDLE	1	0.863621128959242	1	1	1	1	1	1	0.801085314684435
HALLMARK_MTORC1_SIGNALING	1	1	1	1	1	1	1	1	1
HALLMARK_MYC_TARGETS_V1	1	0.866376186700404	1	1	1	1	1	1	1
HALLMARK_MYC_TARGETS_V2	1	1	1	1	1	1	1	1	1
HALLMARK_MYOGENESIS	1	0.59087889947753	0.222936379518387	1	0.411222990169279	0.679156739870076	1	0.333982300763813	0.212763319134831
HALLMARK_NOTCH_SIGNALING	1	1	1	1	1	1	1	1	1
HALLMARK_OXIDATIVE_PHOSPHORYLATION	1	0.128805218718827	1	1	1	1	1	1	1
HALLMARK_P53_PATHWAY	0.385433769980492	0.863621128959242	1	1	1	1	1	0.698962798501376	1
HALLMARK_PANCREAS_BETA_CELLS	1	1	1	1	1	1	1	1	1
HALLMARK_PEROXISOME	1	0.266878054642781	1	1	1	1	1	1	1
HALLMARK_PI3K_AKT_MTOR_SIGNALING	1	1	1	1	1	1	1	1	1
HALLMARK_PROTEIN_SECRETION	0.207515072172784	1	1	1	1	1	1	1	1
HALLMARK_REACTIVE_OXYGEN_SPECIES_PATHWAY	1	1	1	1	1	1	1	1	1
HALLMARK_SPERMATOGENESIS	1	1	1	1	1	1	1	0.554914595429739	0.663405339725604
HALLMARK_TGF_BETA_SIGNALING	1	1	1	1	1	1	1	1	1
HALLMARK_TNFA_SIGNALING_VIA_NFKB	1	0.316384572296279	1	0.203873338499211	1	1	0.455868767341762	1	1
HALLMARK_UNFOLDED_PROTEIN_RESPONSE	1	1	1	1	1	1	1	1	1
HALLMARK_UV_RESPONSE_DN	0.295630318080048	0.761681365941078	0.476729043924512	0.149253492377817	0.314389192538431	1	1	1	1
HALLMARK_UV_RESPONSE_UP	1	0.20269565105404	1	1	1	1	1	1	1
HALLMARK_WNT_BETA_CATENIN_SIGNALING	1	1	1	1	1	1	1	1	1
HALLMARK_XENOBIOTIC_METABOLISM	0.0830335909743674	0.319094270033555	0.600741543344345	1	0.414377851285671	0.682835050127822	0.457537241799894	1	0.807559661524495

Table 5. Table showing the run time of algorithms for calculating gene modules on an Ubuntu DELL XPS 9320 laptop with 32 GB RAM. Note that the calculation of pseudocells (14.75 s) is not included in the scWCNA runtime.

Software	Runtime (s)
scWCNA	110.38
GeneNMF	7.66
Antler	14.81
CatsCradle	0.15

References

Aibar, S., González-Blas, C. B., Moerman, T., Huynh-Thu, V. A., Imrichova, H., Hulselmans, G., Rambow, F., Marine, J.-C., Geurts, P., Aerts, J., van den Oord, J., Atak, Z. K., Wouters, J., and Aerts, S. (2017). SCENIC: single-cell regulatory network inference and clustering. *Nat. Methods*, 14(11):1083–1086.

Delile, J., Rayon, T., Melchionda, M., Edwards, A., Briscoe, J., and Sagner, A. (2019). Single cell transcriptomics reveals spatial and temporal dynamics of gene expression in the developing mouse spinal cord. *Development*, 146(12):dev173807.

Durinck, S., Moreau, Y., Kasprzyk, A., Davis, S., De Moor, B., Brazma, A., and Huber, W. (2005). Biomart and bioconductor: a powerful link between biological databases and microarray data analysis. *Bioinformatics*, 21:3439–3440.

Feregrino, C. and Tschopp, P. (2022). Assessing evolutionary and developmental transcriptome dynamics in homologous cell types. *Dev. Dyn.*, 251(9):1472–1489.

Laddach, A. and Shapiro, M. (2024). CatsCradle. https://bioconductor.org/packages/release/bioc/html/CatsCradle.html.

Moran, P. A. P. (1950). Notes on continuous stochastic phenomena. *Biometrika*, 37(1/2):17–23.

Van de Sande, B., Flerin, C., Davie, K., De Waegeneer, M., Hulselmans, G., Aibar, S., Seurinck, R., Saelens, W., Cannoodt, R., Rouchon, Q., Verbeiren, T., De Maeyer, D., Reumers, J., Saeys, Y., and Aerts, S. (2020). A scalable SCENIC workflow for single-cell gene regulatory network analysis. *Nat. Protoc.*, 15(7):2247–2276.

Yerly, L., Andreatta, M., Garnica, J., Nardin, C., Domizio, J. D., Aubin, F., Gilliet, M., Carmona, S. J., and Kuonen, F. (2025). Wounding triggers invasive progression in human basal cell carcinoma. *bioRxiv*.
